# Artificial Intelligence for Caregivers of Persons With Alzheimer’s Disease and Related Dementias: Systematic Literature Review

**DOI:** 10.2196/18189

**Published:** 2020-08-20

**Authors:** Bo Xie, Cui Tao, Juan Li, Robin C Hilsabeck, Alyssa Aguirre

**Affiliations:** 1 School of Nursing The University of Texas at Austin Austin, TX United States; 2 School of Information The University of Texas at Austin Austin, TX United States; 3 School of Biomedical Informatics The University of Texas Health Science Center at Houston Houston, TX United States; 4 Department of Computer Science North Dakota State University Fargo, ND United States; 5 Department of Neurology Dell Medical School The University of Texas at Austin Austin, TX United States

**Keywords:** Alzheimer disease, dementia, caregiving, technology, artificial intelligence

## Abstract

**Background:**

Artificial intelligence (AI) has great potential for improving the care of persons with Alzheimer’s disease and related dementias (ADRD) and the quality of life of their family caregivers. To date, however, systematic review of the literature on the impact of AI on ADRD management has been lacking.

**Objective:**

This paper aims to (1) identify and examine literature on AI that provides information to facilitate ADRD management by caregivers of individuals diagnosed with ADRD and (2) identify gaps in the literature that suggest future directions for research.

**Methods:**

Following the Preferred Reporting Items for Systematic Reviews and Meta-Analyses guidelines for conducting systematic literature reviews, during August and September 2019, we performed 3 rounds of selection. First, we searched predetermined keywords in PubMed, Cumulative Index to Nursing and Allied Health Literature Plus with Full Text, PsycINFO, IEEE Xplore Digital Library, and the ACM Digital Library. This step generated 113 nonduplicate results. Next, we screened the titles and abstracts of the 113 papers according to inclusion and exclusion criteria, after which 52 papers were excluded and 61 remained. Finally, we screened the full text of the remaining papers to ensure that they met the inclusion or exclusion criteria; 31 papers were excluded, leaving a final sample of 30 papers for analysis.

**Results:**

Of the 30 papers, 20 reported studies that focused on using AI to assist in activities of daily living. A limited number of specific daily activities were targeted. The studies’ aims suggested three major purposes: (1) to test the feasibility, usability, or perceptions of prototype AI technology; (2) to generate preliminary data on the technology’s performance (primarily accuracy in detecting target events, such as falls); and (3) to understand user needs and preferences for the design and functionality of to-be-developed technology. The majority of the studies were qualitative, with interviews, focus groups, and observation being their most common methods. Cross-sectional surveys were also common, but with small convenience samples. Sample sizes ranged from 6 to 106, with the vast majority on the low end. The majority of the studies were descriptive, exploratory, and lacking theoretical guidance. Many studies reported positive outcomes in favor of their AI technology’s feasibility and satisfaction; some studies reported mixed results on these measures. Performance of the technology varied widely across tasks.

**Conclusions:**

These findings call for more systematic designs and evaluations of the feasibility and efficacy of AI-based interventions for caregivers of people with ADRD. These gaps in the research would be best addressed through interdisciplinary collaboration, incorporating complementary expertise from the health sciences and computer science/engineering–related fields.

## Introduction

Alzheimer’s disease and related dementias (ADRD) have become a major public health concern in the United States. An estimated 5.6 million Americans aged 65 and older (10% of the US population) were living with ADRD in 2019, and this number is expected to grow dramatically as the population continues to age. By 2025, the number of Americans aged 65 or older with ADRD is expected to reach 7.1 million, nearly a 27% increase from 2019, and by 2050, this population is projected to be 13.8 million, with the highest growth among those in ADRD’s advanced stage [1].

Persons with ADRD require progressively extensive assistance in their daily lives, the majority of which is provided by family members, friends, and other unpaid caregivers [1]. It is estimated that in 2018, American caregivers of persons with ADRD provided 18.5 billion hours of informal unpaid assistance, valued at $233.9 billion [1]. Family caregivers (hereafter “caregivers”) of persons with ADRD are expected to make important care decisions for their family members with ADRD on a daily basis. However, these caregivers report being unprepared for their roles and responsibilities, uninformed about care options, and unsupported by professionals in their decision making [2-5]. Caregiving for persons with ADRD is stressful [6-10], and it can severely affect the caregiver’s own health and well-being [7]. There is an urgent need to better prepare caregivers to manage their daily lives and those of their family members with ADRD, yet there are critical knowledge gaps regarding the types and amounts of information that caregivers may want to have in order to better manage ADRD. To provide patient-centered care for people with ADRD and enhance caregivers’ quality of life, we must address those gaps.

Artificial intelligence (AI) is showing great promise in areas of health care—in precision treatments, patient education, virtual assistance, and cost reduction [11]. Some attempts have been made to apply AI for persons with ADRD and their caregivers in order to improve patients’ daily functioning, quality of life, and well-being, as well as reduce caregiver burden (eg, social robots to facilitate social interaction and engagement, assistive robots to facilitate daily activities such as handwashing, tea making, or dressing) [12-16]. To date, however, there has been little systematic review to identify research on AI for ADRD management by caregivers and gaps that remain in our understanding of AI for ADRD management. We have conducted this systematic review to identify and examine literature on AI that provides information to facilitate ADRD management by caregivers of individuals diagnosed with ADRD and to identify gaps in the literature that suggest future directions for research.

## Methods

### Overview

Following Preferred Reporting Items for Systematic Reviews and Meta-Analyses (PRISMA) guidelines for conducting systematic literature reviews and following procedures used in previous systematic literature reviews [17-19], we performed 3 rounds of search in selected databases. Because this review focuses on AI and ADRD management, we searched databases commonly used for research not only in the health sciences but also in computer science and engineering: PubMed, Cumulative Index to Nursing and Allied Health Literature (CINAHL) Plus with Full Text, PsycINFO, IEEE Xplore Digital Library, and ACM Digital Library. First, we searched titles and abstracts using keywords. Next, we screened the titles and abstracts using inclusion and exclusion criteria. Finally, we screened the papers’ full texts to ensure that they met the inclusion or exclusion criteria.

### Round 1: Keyword Search

On August 23, 2019, we searched titles and abstracts in PubMed using the following 3 sets of keywords: (“dementia” OR “Alzheimer”) AND (“caregiver*” OR “proxy” OR “proxies” OR “surrogate*”) AND (“artificial intelligence” OR “intelligent”). These sets of keywords were inclusive but in line with our study’s aims. For the same reason, we did not use built-in limiters in PubMed. This yielded 16 papers. Next, we performed the same search of medical subject heading (MeSH) terms in PubMed, excluding “proxies” and “intelligent,” which are not MeSH terms: (“dementia” OR “Alzheimer”) AND (“caregiver*” OR “proxy” OR “surrogate*”) AND (“artificial intelligence”). This yielded 10 papers. Of the 26 papers from these two searches, 1 was a duplicate, yielding a combined total of 25 nonduplicate papers (4 were reviews; the other 21 reported original data). In addition, we searched both CINAHL Plus with Full Text and PsycINFO, using the same 3 sets of keywords that we used for titles and abstracts in PubMed. CINAHL yielded 10 papers, including 7 duplicates. PsycINFO yielded 10 papers, including 6 duplicates. Excluding duplicates, 7 papers remained, for a total of 32 papers across the 3 health sciences databases.

Again, on September 9, 2019, using the same sets of keywords, a search of all metadata (titles, abstracts, and indexing terms) for all available years in the IEEE Xplore Digital Library yielded 47 papers. We also searched the ACM Digital Library (ACM Full-Text Collection) for abstracts or titles that matched any of the following words or phrases: “Alzheimer’s,” “dementia,” “caregiver,” “proxy,” “proxies,” “surrogate,” “artificial intelligence,” “intelligent.” These results were sorted by relevance, and the first 200 records were manually inspected; this generated 36 papers. No duplicates were found between the ACM and IEEE databases. However, when merged with the first 32 papers from PubMed, CINAHL, and PsycINFO, 2 duplicates were found, yielding a total of 113 nonduplicate papers.

### Round 2: Screening of Titles and Abstracts

Next, 3 of the authors (BX, CT, JL) each screened approximately one-third of the titles and abstracts of the 113 papers. The results were cross-examined by the other 2 authors to ensure accuracy and consistency. Differences were resolved through several rounds of discussion. This round of screening was based on the rationale that the focus of our systematic literature review was AI tools that could provide information and service to facilitate ADRD management by caregivers of persons diagnosed with ADRD. Other topics were outside of the scope of our review. Specifically, we removed any paper that met at least one of the following exclusion criteria: (1) primary focus on using artificial intelligence to automatically collect information from users (eg, via sensors), not to provide information to users (n=32); (2) paper did not report empirical data from human participants (eg, literature review, book review, column/commentary, system architecture; n=9); (3) primary focus on screening, identification, or diagnosis of dementia or detecting or modeling anxiety or burnout in caregivers instead of providing services to persons already diagnosed with dementia or their caregivers (n=5); (4) study participants were paid or volunteer caregivers and did not include any family caregivers (n=3); (5) full text not in English (n=3).

This round of screening resulted in the removal of 52 papers, with 61 papers remaining.

### Round 3: Screening of Full Text

In the next round of screening, we eliminated 31 more papers because they met at least one of the aforementioned exclusion criteria: (1) did not report empirical data from human participants (n=18); (2) study participants did not include family caregivers (n=4); (3) primary focus on using artificial intelligence to automatically collect information from users (eg, via sensors), not to provide information to users (n=4); (4) technology under investigation was not artificial intelligence (eg, videogames; n=3); (5) primary focus on screening, identification, or diagnosis of dementia or detecting or modeling anxiety or burnout in caregivers (n=1); (6) report of essentially the same content as in another paper (n=1).

**Figure 1:**
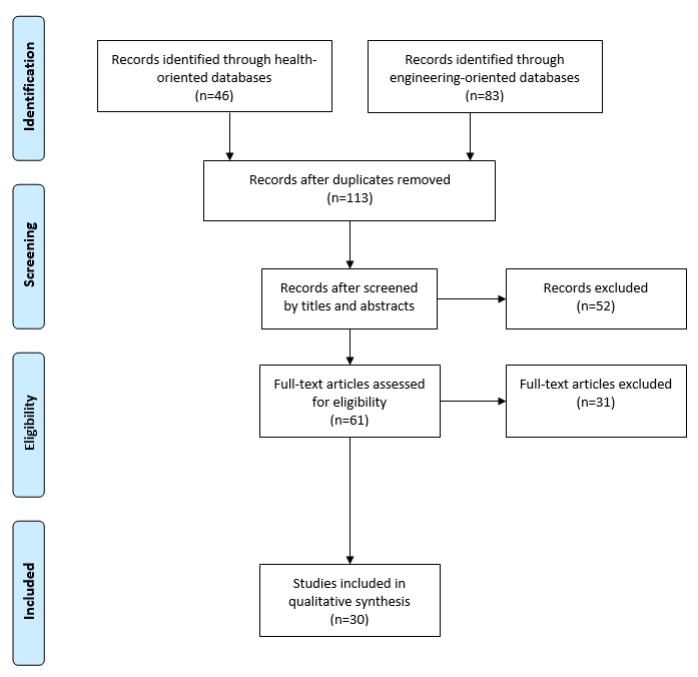
Search and screening process.

A total of 30 papers remained in the final sample [20-49]. The selection process is summarized in Figure 1 according to the PRISMA guidelines [50].

### Round 4: Coding of Full Text

The 30 papers in our final sample were coded using a framework consistent with our prior work [17-19], summarizing key information from each paper. The coding included each study’s publication year, study aim, research method, participant characteristics, sample size, country/area where data collection took place, dosage of AI technology (ie, amount and frequency of time exposed to the AI technology), outcome measures, and key findings. The results of the coding are presented in Multimedia Appendix 1. In addition, we assessed levels of evidence reported in the 30 papers [51].

## Results

Our initial searches yielded 113 papers. Through multiple rounds of screening, we removed 83 of them to arrive at our final sample. The reasons for excluding these 83 papers are summarized in Table 1.

**Table 1:** Summary of the reasons for excluded papers.

Reason for exclusion	n^a^
Primary focus was using AI^b^ to automatically collect information from users (eg, sensors), not to provide information to users	36
Did not report empirical data from human participants (eg, literature review, book review, column/commentary, system architecture)	27
Study participants did not include any family caregivers	7
Primary focus was on screening/identification/diagnosis of dementia or detecting/modeling anxiety or burnout in caregivers (instead of providing services to persons already diagnosed with dementia or their caregivers)	6
The technology under investigation was not AI	3
Full text not in English	3
Reporting essentially the same content as another paper (that was already included in the final sample)	1
Total	83

^a^Number of excluded papers.

^b^AI: artificial intelligence.

Key characteristics of the final 30 papers are summarized in Multimedia Appendix 1. The papers were published from 2001 to 2019, averaging 2 per year. The number of publications was consistently low (1 per year) until rising in 2008. The year 2018 had the most papers (4), suggesting an increasing interest in our topic.

AI technologies included in the 30 papers varied. We categorized these technologies according to their intended use. As Table 2 shows, the majority (20/30, 67%) focused on using AI to assist in activities of daily living. A limited number of specific daily activities were targeted in these studies, particularly handwashing, tea making, and dressing.

**Table 2:** Summary of artificial intelligence technology’s intended use.

AI^a^ technology use	n^b^
Assist in activities of daily living (eg, assistive robots to aid handwashing, tea making, or dressing)	20
Facilitate social interaction (eg, social robots)	2
Provide cognitive stimulation (eg, computerized activities to stimulate cognition)	2
Ensure safe home environments (eg, smart homes)	2
Educate (eg, through a teleconferencing program or virtual reality platform)	2
Assist in reminiscence therapy	2
Total, N	30

^a^AI: artificial intelligence.

^b^Number of papers.

Aims of the 30 studies fell into one of three major categories: (1) to test the feasibility, usability, or perceptions of a prototype AI technology; (2) to generate preliminary data on the technology’s performance (primarily accuracy in detecting target events, such as falls); and (3) to understand user needs and preferences for the design and functionality of to-be-developed technologies.

The majority of these studies used qualitative research methods, with interviews, focus groups, and observation being the 3 most common methods. Cross-sectional surveys were also common, but with small convenience samples. The majority of the studies were descriptive, exploratory, and lacking in theoretical guidance; they were not intended to test theory-informed hypotheses.

The sample sizes of all 30 studies were small, ranging from 6 to 106, with the vast majority on the low end. A total of 7 studies reported data from healthy volunteers or health care professionals but did not include actual patients or caregivers as research participants. We included these studies in our analysis because the technologies under consideration were intended for use by patients and caregivers. One study did not report any participant characteristics.

Nearly half of the studies were conducted in Canada (14/30, 47%), 8 of the 30 (27%) in Europe, and 4 of the 30 (13%) in the United States. Israel, Japan, Mexico, and Taiwan each had one study (1/30, 3%). At least 7 of the 30 studies (23%) were conducted in a research lab; 6 others did not report the setting for data collection. The remaining studies took place in a facility (eg, senior living facility, hospital) or private home.

We also analyzed the AI technology’s dosage (ie, the amount of time and frequency that users were exposed to the AI technology in each study). A total of 9 of the 30 studies used interviews or surveys, so the dosage criterion was not applicable to them. Among the 21 studies that involved exposure to AI technology, 5 did not report dosage. The dosages reported in the remaining 16 studies varied widely in terms of both total time and frequency of exposure, ranging from as much as 24/7 access for 4 to 6 weeks or 2 hours per week over 12 weeks to as little as 15 to 20 minutes in a single session.

Outcome measures varied widely as well. Overall, they included both objective and subjective measures. Outcome measures included (1) feasibility, satisfaction, and stress, which were subjective measures; (2) performance, such as the accuracy of AI technology in completing its intended task, measured objectively; (3) usability (self-reported ease of use and perceptions of usefulness); (4) usage patterns (eg, which AI features were used, frequency/duration of usage), also measured objectively; and (5) user needs and requirements for the technology, another set of subjective measures.

Many studies reported positive outcomes in favor of the AI technology being studied (or to be developed) in terms of the technology’s feasibility (with acceptability used as the most common measure of feasibility) and satisfaction (positive perceptions of the technology). One of those studies reported a high dropout rate (65%) [22], making it difficult to interpret the study’s reported positive outcomes. One study reported preliminary evidence supporting limited efficacy of a social robot in reducing patients’ stress [29]. Some studies reported mixed results for feasibility and satisfaction, with some participants reporting that they liked the AI technology but others reporting that they did not [23,26,47,49]. Notably, in 2 separate studies, caregivers reported more positive attitudes than did patients toward the use of AI technology in home care [23,49].

Performance of the technology, measured primarily by accuracy in detecting target events, varied widely across different tasks, ranging from as low as 23% in detecting incorrect dressing events [28] to as high as 98% in detection of falls [46]. In assisting with daily activities, assistive AI devices helped reduce patients’ dependence on caregivers [42,43]. Usage patterns also varied widely, ranging from continuous active use to inactive use. Mixed results were reported for the AI technology’s features, with some features easier to use and more popular than others [33].

A range of user needs was identified, including needs for assistance in home care, getting information (about time, schedule, care options, etc), and communication and social interactions. There is a great need for AI technology to provide tailored assistance to meet these user needs [37]. However, several factors make it challenging to design tailored technology. These include variation in patients’ needs and abilities from day to day and even during the day [39], patients’ varying and evolving identities and preferences for a technology’s styles and features [40], users’ diverse technology literacy levels [41], and challenges associated with ethical issues [48], particularly conflicting needs between caregivers and patients [36] and privacy concerns in assisting in private tasks [31,32].

Regardless of the findings, the levels of evidence [51] of all studies in our final sample were low due to their small convenience samples and exploratory research methods.

## Discussion

AI has great potential for improving the care for persons with ADRD and the quality of life of family caregivers. To date, however, there has been little effort to systematically review literature on AI for ADRD management by caregivers and to determine what still needs to be done to understand the impact of AI on ADRD management. In this study, we have addressed those gaps. We have identified work on AI that provides information to facilitate ADRD management by family caregivers of patients diagnosed with ADRD, and we have identified gaps in existing work, which suggest future directions for research. The majority of the AI studies included in our final sample (20/30, 67%) focused on using AI to assist in activities of daily living. A limited number of specific daily activities were targeted. The aims of the 30 studies suggested three major purposes: (1) to test the feasibility, usability, or perceptions of a prototype AI technology; (2) to generate preliminary data on the technology’s performance (primarily accuracy in detecting target events, such as falls); and (3) to understand user needs and preferences for the design and functionality of to-be-developed technologies. The majority of these studies used qualitative research methods, with interviews, focus groups, and observation being the 3 most common methods. Cross-sectional surveys were also common, but with small convenience samples. The sample sizes of the 30 studies were small, ranging from 6 to 106, with the vast majority on the low end. The majority of the studies were descriptive, exploratory, and lacking in theoretical guidance. Many studies reported positive outcomes in favor of AI technology’s feasibility and user satisfaction; some reported mixed results for these measures. Performance of technology varied widely across different tasks.

Our findings illustrate important characteristics of research to date on the use of AI that provides information to aid ADRD management by family caregivers. First, only a few studies (N=30) have focused on this topic. Given the topic’s interdisciplinary nature, we intentionally searched databases commonly used in the health sciences and in computer science/engineering. We found only 2 duplicates between these 2 sets of databases, with more than two-thirds of the studies in the computer science/engineering databases (32 from the health sciences databases, 83 from the computer science/engineering databases). On the topic of AI in ADRD management by caregivers, there was little overlap between the health sciences and computer sciences/engineering databases, suggesting that the latter databases currently contain the majority of existing research. To review developments on this topic, one must examine both sets of databases. Future systematic literature reviews should also track potential changes in the ratio of work found between these sets of databases as an indicator of the maturity of the technology and its applications in health care. It is likely that, as time goes by, when AI technology and its applications in health care are more mature, the research found in the health sciences databases will increase, while that in computer science/engineering databases may decrease (in absolute number or relative ratio).

We also found that a large number of studies (n=36) had the primary focus of using AI to automatically collect information from users (eg, via sensors), which would be used by health care professionals to make care decisions. We did not include those studies in our final sample because our review was meant as a basis for the development of AI-based interventions to provide information to family caregivers (our interdisciplinary team is currently working on such an intervention). However, acknowledging that collecting user information is necessary for providing tailored information, we did include studies that both collected information from and provided information to caregivers. It was beyond the scope of the present review to include studies that focused only on collecting information. Researchers interested in obtaining a full list of those studies may contact the first author for that list.

We also found a large number of papers (n=27) that did not report empirical data from human participants. Some were common types of papers reporting nonempirical data (eg, literature reviews, book reviews, and columns/commentaries), which one would typically expect from searches of health sciences databases (as in prior reviews [17-19]). Characteristically for the present systematic literature review, however, we also found a number of nonempirical studies reporting technical specifics or system architecture for designing AI systems. This is typical of technology development–related work commonly reported in computer science/engineering databases but uncommon in health sciences databases. Further, of the studies that did report empirical data, the majority were descriptive, exploratory, with small convenience samples, and lacking theoretical guidance. Such studies have their own merit and are appropriate for the current stage of research. However, they also show that research on AI for ADRD management is still in the stage of technological development and far from ripe for clinical evaluation. It is premature at this point to systematically examine the efficacy of AI interventions for patients and caregivers.

Consistent with the early stage of research in this area, the aims of the 30 studies in our sample focused on testing the feasibility, usability, or perceptions of prototype AI technologies; generating preliminary data on the technology’s performance; and understanding user needs and preferences for the design and functionality of to-be-developed or to-be-revised technologies. Key study findings showed mixed results. Some studies reported promising signs for the acceptability and feasibility of AI tools, but others found challenges that must be addressed before large-scale rollout of AI tools for ADRD management. Notably, the studies in our sample frequently did not report key pieces of information necessary for extraction in health science–oriented systematic reviews, including research participants’ demographics, research settings, or even locations where data collection took place. Many of the studies may have been conducted by researchers with training in non–health science fields, such as engineering and computer science, in which reporting norms differ from those commonly used in the health sciences. As a result, systematic review methods and quality criteria commonly used in the health sciences, such as levels of evidence [51], are not easily applicable to current research on AI for ADRD management. This presents an opportunity for interdisciplinary collaboration between researchers in the health sciences and in computer science/engineering–related fields (as is the case for our interdisciplinary team, with expertise in nursing, medicine, and social work on the one hand and in computer science and informatics on the other).

Our systematic review has limitations. We selected only papers with full text in English, so we might have missed cutting-edge studies in other languages. The selection of our initial search terms was also not exhaustive; AI is a broad concept that includes technologies that may be labeled under different terms but are nonetheless still AI based. By using only “artificial intelligence” or “intelligent” as our AI-related search terms, we might have missed technologies that did not use these terms but did use AI (eg, expert systems, decision aids). However, a merit of our approach is that it allowed us to focus on publications self-labeled by their authors as AI-related work. By using “artificial intelligence” and “intelligent” as our AI-related search terms, we were able to focus on studies defined by their authors as reporting AI-related technology and thus to identify researchers who self-identify as AI researchers. Overall, our review has identified work on AI that provides information to facilitate ADRD management by caregivers, as well as gaps in the literature that require future research. These findings call for more systematic designs and evaluations of the feasibility and efficacy of AI-based interventions for caregivers. Such tasks will be best addressed through interdisciplinary collaboration incorporating complementary expertise from the health sciences and computer science/engineering–related fields.

## Appendix

Multimedia Appendix 1 Summary of the 30 studies in the final sample.

## Abbreviations

ADRD: Alzheimer’s disease and related dementias
AI: artificial intelligence
CINAHL: Cumulative Index to Nursing and Allied Health Literature
MeSH: medical subject heading
PRISMA: Preferred Reporting Items for Systematic Reviews and Meta-Analyses
