# Supplementary material for: Artificial Intelligence for Caregivers of Persons With Alzheimer’s Disease and Related Dementias: Systematic Literature Review
Source: JMIR Med Inform. 2020 Aug 20;8(8):e18189. doi: 10.2196/18189 (PMC7471889; doi:10.2196/18189)
Supplement: Multimedia Appendix 1 [file medinform_v8i8e18189_app1.docx]

Multimedia appendix. summary of the 30 studies in the final sample

| Author | Year | Study Aim | Method | Participant | Country/Area | Setting | Dosage | Outcome Measure | Key Finding |
| --- | --- | --- | --- | --- | --- | --- | --- | --- | --- |
| Abdollahi et al.^20^ | 2017 | To generate pilot data on the feasibility of a social robot (usage; perception) | Analysis of usage data auto-collected from logs; survey of users; interview of 1 nurse | 6 older adults with early to moderate dementia and/or depression; age range: 63-86 | U.S. | Senior living facility | 24/7 access for 4-6 weeks | Interaction pattern (conversation topics, number of dialogs, etc.); use of AI activities/features (that were not conversational); likability; acceptance | Active, continuous use of the tool; positive perception |
| Amiribesheli et al.^21^ | 2015 | To validate a user-centered approach to designing smart homes for PWDs | Interviews | 4 social caregivers and 2 dementia specialists | UK | Not reported | n/a | Participants' feedback on the design scenarios | Scenario-based design approach validated (but very little empirical data reported) |
| Apostolidis et al.^22^ | 2014 | To test the feasibility of a teleconferencing educational program for ADRD caregivers | Not specified | 60 (21 completes), age range: 20-65 | EU | Remote | 24 distance lessons over 12 weeks, 2 hr per session, plus 2 1-hr personal sessions with a psychologist | Satisfaction and use | Seemed positive among completers, although dropout was 65%; very little empirical data; mostly on design/content of educational program |
| Begum et al.^23^ | 2013 | To assess the feasibility and usability of an assistive robot to help PWDs perform ADLs | Semi-structured interviews; survey | 5 patients plus 5 family caregivers; age range: 59-88 | Canada | Simulated home setting (in a research lab) | Not reported (presumably 2 hr for 1 session?) | Feasibility (qualitative); Usability (effectiveness; satisfaction) | Caregivers all positive; patients mostly positive, 1 patient felt not easy to use, perceived no usefulness with no acceptance |
| Begum et al.^24^ | 2015 | To identify design challenges for assistive robots to help PWDs in ADL or social interaction (a tea-making task was tested) | Observation; interviews | 10 PWDs and family caregivers; 59-88 | Canada | Simulated home setting (in a research lab) | 2.5 hr | Interaction behaviors (Interactiveness; natural dialogue; team behavior; emotion; cognition and memory) | Some level of trust, grounding, communication, and situational awareness, but more improvement needed |
| Berenbaum et al.^25^ | 2011 | To understand the benefits of computerized stimulation activities for Alzheimer's patients | Thematic analysis of 80 reports randomly selected from 10 years' reports (comments left on a therapy evaluation form by patients who used the computer system and therapists who carried out the therapy sessions) | Patients and activity workers; sample size and participants' demographics not reported | Israel | Adult daycare centers | Each session is about 20-30 min, with one therapy evaluation form filled out (by a computer activity worker) | Patients' ability to learn to use computer system; feedback | Alzheimer's patients were able to learn to use a computer system that provided cognitive stimulation and communication with family; feedback mostly positive |
| Berezina-Blackburn et al.^26^ | 2018 | To collect feedback on a virtual reality platform designed for users to simulate and learn about the experiences of dementia patients and caregivers | Focus group | Not actual dementia patients or caregivers; 14 participants immersed in the simulation, another 40-50 observed and engaged in follow-up discussions; participants' demographics were not reported | U.S. | Research lab | 15-20 min | Emotions; attitudes | Mixed emotional reactions; increased awareness, understanding, and empathy for dementia patients and caregivers |
| Boger et al.^27^ | 2006 | To evaluate the efficacy of a computerized planning system (vs. a human-guided system) that uses Markov decision processes (MDP) to guide dementia patients' daily activities (handwashing as the example tested) | Survey (participants completed a questionnaire after watching a video clip); interviews | 30 professional caregivers | Canada | Long-term care facilities | n/a | Effectiveness of prompts to guide patient to complete handwashing activity | Computerized system effective, suggesting that MDP can be applied to guide design of such a system; however, the computerized system was less effective than a human-guided system |
| Burleson et al.^28^ | 2018 | To assess the capability of a prototype intelligent system, the DRESS prototype, in detecting correct and incorrect dressing events that may aid PWDs’ dressing activity | Experiment with pre- and post-surveys | 11 healthy participants age 19-41 (mean age: 25) | U.S. | Research lab | 1 hr | Accuracy in detecting correct and incorrect dressing events simulated by healthy young adults | Prototype incorrectly identified 10/22 cases for shirts and 5/22 cases for pants |
| Chan et al.^29^ | 2011 | To assess the ability of a social robot, Brian 2.0, to engage PWDs in a cognitively stimulating game while minimizing their stress | Experiment | 6 healthy adults, all had past experience with the game (age 21-35) | Canada | Research lab | Not reported | Stress | Socially intelligent robot promising in reducing stress during game play |
| Chen^30^ | 2009 | To assess the usage and perceptions of a mobile guiding and tracking system in public transit systems | Field experiment | 6 patients recruited from 5 rehabilitation hospitals, ages 21-44 | Taiwan | Hospital | Not reported | Satisfaction and use | Mobile system easy to use, perceived as useful |
| Czarnuch, Mihailidis^31^ | 2011 | To understand the needs of PWDs and their family caregivers during ADL and how intelligent assistive technology can support their needs | Survey | 106 current, primary, informal caregivers of PWDs age 21-77 (mean age: 56) | Canada | Survey both online and paper-based | n/a | ADLs that are challenging to PWDs; ADLs that are difficult for caregivers to assist; the roles, features, and functions of in-home assistive technology | PWDs had partial ability to complete ADLs; private tasks were challenging to assist; assistive technology must be autonomous and easy to use; few participants were aware of existing technology that might meet their needs |
| de Jong et al.^32^ | 2018 | To assess the needs of end-users and caregivers for a virtual agent "Anne" designed to support PWDs' independent living at home and their caregivers | Focus groups | No PWD; Italy: 3 professional and 3 informal caregivers; Luxembourg: 6 qualified nursing assistants and 4 informal carers | EU | Two health-related organizations | Each focus group lasted about 120 min | Needs for (1) independent living at home, (2) caregiving, and (3) the virtual agent's (current and future) features; Quality of life | Virtual agent may be useful and have a positive impact; some concerns identified (e.g., privacy); further research needed |
| Edmeads et al.^33^ | 2019 | To understand how technology can be used to assist PWDs to engage in reminiscence therapy | Participatory design; semi-structured interviews; survey | 5 PWDs age 86-94; professionals and caregivers (number of the second group not reported) | UK | A medium-sized nursing home (20 residents) | n/a | Needs and requirements for technology; use and perceptions of the prototypes | Mixed results: some prototype features easy to use and popular, others not |
| Hawkey et al.^34^ | 2005 | To evaluate the feasibility of an information appliance (i.e., a computerized device designed to provide information) to reduce PWDs’ reliance on caregivers for information and thus reducing repetitive questioning behaviors common among PWDs (which, subsequently, may reduce caregiver stress) | Semi-structured interviews (with PWDs, caregivers) and diaries (recorded by caregivers) | Seven pairs of PWD and caregivers | Canada | Home (6/7 pairs) Clinic (1 pair) | Caregiver interviews lasted about 45-60 min each; interviews with PWD lasted about 20-30 min each | PWDs’ information needs, preferences for communication modes, repetitive questioning patterns | PWDs need information about time, schedule, current event details, reminders, and opinions; no PWD currently able to use a computer, with difficulty using common household devices like TV remote, radio, telephone; repetitive questioning occurred daily and caused stress; current strategies in coping with repetitive questioning, e.g., keeping a journal, diary, or message board, were ineffective |
| Horwitz et al.^35^ | 2008 | To understand the home-care needs of PWDs and their informal caregivers and whether or not commercially available and in-development technology can meet those needs | Survey; focus group | Surveyed patients with heart disease and dementia; focus group included patients and informal caregivers; sample size and participant demographics not reported | U.S. | Not reported | n/a | Patients' and informal caregivers' needs for home-care technology | Patients and family caregivers need intelligent technology to support ADRD care at home |
| Hwang et al.^36^ | 2012 | To understand the needs of informal caregivers for the user interface design of an intelligent system, COACH | Phase 1, Participatory design; Phase 2, Usability testing with a paper prototype | Phase 1, 6 informal caregivers (ages 37-77); Phase 2, 2 participants from Phase 1 | Canada | Phase 1 setting, not reported; Phase 2, participants' private homes | Phase 1, two 90-min sessions, 1 month apart; Phase 2 time not reported | Participants' feedback on the prototype, and their recommendations for intelligent home system's user interface design | Participants generally positive about the system; conflicting needs identified, presenting design challenges |
| Hwang et al.^37^ | 2015 | To explore how technologies for ambient assisted living (AAL) might aid informal caregivers to care for PWDs | Multiphase codesign including Phase 0, design preparation; Phase 1, concept development; Phase 2, concept refinement; Phase 3, user trials; Phase 4, concept interpretation | 6 caregivers of persons with mild dementia (ages 37-77) participated in Phases 1 and 2, and 2 of them participated in Phase 3 | Canada | A boardroom of a community-based agency serving PWDs and caregivers | Phases 1 and 2, 90 min each; Phase 3, 2 hr | Participants' feedback on design of the prototypes and their requirements for the technology | AAL technologies should be designed to complement caregivers' care strategies, enable caregivers to develop trust and confidence in the technology's abilities over time, and facilitate provision of personalized care |
| Jean-Baptiste, Mihailidis^38^ | 2017 | To evaluate, and compare, the impact of two task-modeling approaches on the ability of an intelligent assistive technology, COACH, in providing prompts to guide older PWDs to complete a daily activity (handwashing) | Analysis of video recordings of PWDs’ handwashing activities as guided by the system based on the original task modeling and the new one | 20 PWDs (MMSE scores from 9 to 28); no demographics reported | Canada | A washroom installed with the intelligent system (unclear if the washroom was in a research lab or another setting) | Not reported | The system’s ability to correctly provide prompts for completing handwashing activity: accuracy, sensitivity and specificity | New task modeling approach superior to original approach |
| Klein, Uhlig^39^ | 2016 | To understand the communication and social needs of PWDs and how reminiscence therapy via multimedia and multimodal stimuli-based technology may help meet those needs (this 2-page short paper reports an "early" study—it does not have any AI or technology at all; it is an observational study to understand needs—in preparation for future AI development) | Contextual inquiry (mostly observation with few direction interactions) | "about 50” PWDs (exact sample size and demographics not reported) | EU | 2 nursing homes | n/a (amount of contextual inquiries conducted was not reported) | PWDs’ behaviors and interactions with other PWDs and professional caregivers at nursing homes | PWDs’ mood, activity level and motor skills varied from day to day and even during the day, suggesting the time of the day matters to the use of an intervention; technology could be designed to fill in the gaps when caregivers are not available to assist the PWD; caregivers' involvement is important for technology adoption and use |
| König et al.^40^ | 2017 | To understand PWDs’ affective identities as defined by Affect Control Theory (how PWDs feel about their own identities) and their perceptions of a prototype intelligent cognitive assistant (findings will be used in the next phase of a larger study to guide the development of an emotionally intelligent cognitive assistant that can provide tailored prompts to guide PWDs to complete ADL more independently) | Semi-structured interviews | 12 PWDs who reside in care facilities (7 females; mean age, 84.5; age range, 63-96) and 9 caregivers (7 females) | Canada | Not reported | 45 min | Life experience and identities formed, and perceptions of intelligent cognitive assistants | Each participant had multiple identities; some fade, others persist; their identities vary, so do their preferences for the technology's style and features, making it challenging to tailor the technology to individuals’ preferences |
| Lopez et al.^41^ | 2018 | To assess the acceptance of cognitive games delivered through smart TV applications among patients with Parkinson’s Disease as well as professionals and caregivers, and their needs and requirements for the technology's design and functionalities | Individual testing with PWDs, focus groups with health professionals, and collective testing with caregivers | Health professionals, 11; Caregivers, 9; Patients, 16 | EU | Not reported | testing sessions for caregivers and patients: 45 min/  session; health professional engaged in 1 focus group session (duration not reported) | Acceptance and perceptions (e.g., ease of use) of smart TV-based cognitive games among caregivers, professionals, patients | Positive acceptance of smart TV application and cognitive games among patients, caregivers, and health professionals; patients able to learn to use the technology, however patients with less technology experience had more difficulties learning to use it; adaptations needed (e.g., level of the games) for the patient population |
| Mihailidis et al.^42^ | 2004 | To assess the efficacy and effectiveness of an intelligent cognitive assistant, COACH, in guiding PWDs to complete an ADL (handwashing) with less dependence on caregivers | A single subject research design (SSRD), efficacy study | 10 participants with moderate to severe dementia, all male (1 dropped out after baseline; data from 9 were analyzed) | Canada | A test washroom in a health sciences center's long term care unit | 60 days testing/ participant | The rate of completing handwashing without caregivers and the number of times when caregiver guidance was required for PWD to complete handwashing task | Device use reduced dependence on caregivers in all but 1 PWD |
| Mihailidis et al.^43^ | 2008 | To examine the efficacy of a computerized device, COACH, designed to provide prompts that can assist PWDs completing ADL with less dependence on caregivers (thus reducing caregiver burden) | A single-participant research design | 6 older adults with moderate to severe dementia | Canada | a long-term care facility, washroom | One trial session per day on weekdays for 8 weeks, totaling 40 trials per participant | Improvements in completing handwashing with less dependence on a caregiver | With the assistance of the device, participants completed handwashing task more independently, with less dependence on caregivers |
| Mihailidis et al.^44^ | 2001 | To report the system architecture and development process of a computerized device, COACH, to provide prompts that can assist PWDs completing ADL with less dependence on caregivers (thus reducing caregiver burden), and to generate preliminary data (from healthy participants who simulated target behaviors) on the prototype's performance (accuracy, efficacy) that could be used to improve the prototype | Not reported | Not reported | Canada | Not reported | n/a | To test prototype's performance (accuracy, efficacy) and things to improve | Accuracy relatively low (84%); efficacy (i.e., how well the device did what it was designed to do) was 95% |
| Navarro et al.^45^ | 2014 | To understand PWDs’ problematic behaviors and their impact on caregivers in order to develop an ontological model to tailor an ambient-assisted intervention system for the care of PWDs, and to evaluate the acceptance, use, and effects of the intervention | Ontology development phase: contextual interview and observation; Intervention evaluation phase: interviews and diaries | For the ontology development phase, 5 dyads living in the same home (contextual interviews), and 5 residents of an assisted living facility (observation); for the intervention evaluation phase, 1 PWD (log data) and 1 caregiver (pre- and post-intervention interviews) | Mexico | Ontology development phase: private home and assisted living facility; Intervention evaluation: private home | 8 weeks | Ontology development phase: PWDs’ problematic behaviors and their impact on caregivers; Intervention evaluation phase: acceptance/adoption, depression, apathy, independence | Ontology development phase: PWDs’ problematic behaviors were diverse, requiring personalized intervention evaluation phase: positive findings in acceptance/adoption, depression, apathy, and independence |
| Osamu et al.^46^ | 2014 | To develop and evaluate an intelligent device designed to monitor the activities of PWDs (specifically, falls) and generate alerts for caregivers | Not reported | 6 volunteers; no participant characteristics reported beyond simply stating "6 volunteers" | Japan | Mock-up bedroom (likely in a research lab) | Not reported | Accuracy in fall detection; delay in displaying alerts | Fall detection accuracy 98% with 0.03% false positive rate; delay in display, 0.6 s, suggesting high efficacy in monitoring and altering |
| Rialle et al.^47^ | 2008 | To understand ADRD family caregivers' perceptions of technology in aiding home care for PWDs | Survey | 270 family caregivers of PWDs | EU | n/a | n/a | Perceptions of technology in aiding ADRD care | Two opposite clusters of caregivers identified: those in favor of substantial use of technology for ADRD home care, and those opposed |
| Simão & Guerreiro^48^ | 2019 | To design an assistive robot for PWDs and revise based on feedback from stakeholders during an iterative, incremental design process | Interviews | 8 in stage 1 (4 professionals, 2 caregivers, and 2 PWDs); another 18 added later, totaling 26; no participant demographics reported | UK | not reported | n/a | n/a | The assistive robot MATY was well received; modifications were suggested and implemented; ethical issues raised |
| Wang et al.^49^ | 2016 | To understand the perceptions of PWDs and their caregivers for robots that provide prompts for completing ADL in home settings | Observation; interviews | 10 dyads | Canada | Research lab | 5 months | Perceptions of technology in aiding ADRD care | Both PWDs and caregivers reported positive perceptions of robot, but caregivers seemed to have more interest in using the robot than the PWDs |

Acronyms: ADL, activities of daily living; ADRD, Alzheimer’s disease and related dementia; PWD, person with dementia.
